# Cavo-portal transposition in rat: a new simple model

**DOI:** 10.1186/1471-2482-7-18

**Published:** 2007-08-16

**Authors:** Stefano Di Domenico, Giulio Bovio, Maximiliano Gelli, Ferruccio Ravazzoni, Enzo Andorno, Damiano Cottalasso, Umberto Valente

**Affiliations:** 1 Department of General Surgery and Organ Transplantation, S. Martino Hospital, Genoa, Italy; 2 Interventional Radiology, S. Martino Hospital, Genoa, Italy; 3 Department of General Pathology, University of Genoa, Italy

## Abstract

**Background:**

Liver transplantation in presence of diffuse portal vein thrombosis is possible by using caval blood as portal inflow, through cavo-portal transposition. However, clinical results are heterogeneous and experimental studies are needed, but similar hemodynamic conditions are difficult to obtain, especially in small animals. Herein we describe a new simple model of cavo-portal transposition in rat.

**Methods:**

Spontaneous porto-systemic shunts are induced by subcutaneous transposition of the spleen. The presence of porto-caval shunts through the spleen permits the interruption of the main portal vein without splanchnic hemodynamic consequences. Cavo-portal transposition is achieved by anastomosing the inferior vena cava and the main portal vein after division of the pancreatic-duodenal vein.

**Results:**

Selective angiography revealed total splanchnic blood diversion to the systemic venous circulation through the neoformed collaterals; macroscopical examination showed the absence of any signs of acute portal hypertension with normal liver and gut appearance.

**Conclusion:**

This model of cavoportal transposition is simple, effective and it simulates the clinical hemodynamic condition since the porto-systemic shunts induced by splenic subcutaneous transposition correspond to the physiological inframesocolic collaterals during chronic portal thrombosis in man.

## Background

Thrombosis of the portal vein has been a formidable challenge in liver transplantation and it was historically considered an absolute contraindication [1]. Refinements in surgical techniques in the last decades allow surgeons to overcome portal thrombosis with progressive extensions, by using phlebothrombectomy, venous jump grafts or portal vein arterialization [2,3]. Moreover, the use of caval blood as unique portal inflow has been used recently to performed liver transplantation in patients with diffuse splanchnic thrombosis: in this setting graft portal flow has been obtained by end-to-end or end-to-side porto-caval anastomosis or by reno-portal anastomosis [4-6].

Patients with chronic thrombosis without portal hypertension are the best candidates due to the presence of wide spontaneous porto-systemic shunts that completely supply the splancnic vein drainage [7].

The results of these techniques are still heterogeneous and further experimental and clinical studies are needed to clarify the role of spontaneous porto-systemic shunt on liver function after cavoportal transposition [8].

However, similar hemodynamic conditions are difficult to obtain, especially in small animals [9]; herein we described a new simple model of cavoportal transposition in rat with previously induced porto-systemic shunts.

## Methods

Eight male Sprague Dawley rats weighting 250–350 gr were used. Animals were housed for at least 6 days in a light and temperature controlled room (Laboratory of General Pathology, University of Genoa, Italy). Rats were provided access to rodent chow and water ad libitum.

All surgical procedures, including radiological investigation, were performed under general anesthesia after 12 hour fasting with free access to water.

All animals received humane care: Guiding Principles in the Care and Use of Laboratory Animals were strictly adhered to all times together with the recommendations from the Declaration of Helsinki.

Rats underwent subcutaneous transposition of the spleen. After four weeks, angiography and macroscopical examination were performed in four rats (G1) and cavo-portal transposition in the other four (G2). Selective superior mesenteric vein angiography and macroscopical examination were performed in G2 group, 24 hours after cavoportal transposition.

### Anesthesia

General anesthesia was induced by intraperitoneal injection of Zoletil^® ^(Tiletamina + Zolazepam) 80 mg/Kg.

### Splenic transposition

Porto-systemic shunts were induced by subcutaneous spleen transposition [10]. After 1 cm left subcostal incision, the spleen was mobilized and the abdominal wall was sutured behind the spleen with 4/0 silk interrupted stitches between splenic vessel pedicles, that were left intact. After creation of a subcutaneous pouch, the spleen was decapsulated by scoring the surface with needle, and the skin was then sutured in continuos manner.

### Cavo-portal transposition

Four weeks after splenic transposition, cavo-portal anastomosis was performed in G2.

After middle-line incision, the inferior vena cava and portal vein were exposed by gently retraction of gut on the left side; the bowel loops were exteriorized and covered with saline-soaked gauze in order to prevent dehydration.

Portal vein was completely dissected and the pancreatic-duodenal vein was cut between 6/0 silk ligatures; the spleno-mesenteric confluence was then identify. Infrahepatic vena cava was completely dissected above the left renal vein. In order to facilitate the porto-caval anastomosis, the right kidney was fully mobilized.

Portal vein was clamped and cut after 6/0 silk ligature above the spleno-mesenteric confluence. Right kidney vessels and the inferior vena cava were clamped; the infrahepatic vena cava was cut after 6/0 silk ligature.

End-to-end cavo-portal anastomosis was then performed using 10/0 Prolene in continuos manner and the liver was then reperfused.

### Radiological investigation

After general anesthesia induction, porto-systemic shunt formation was evaluated four weeks after splenic transposition by direct spleen angiography and macroscopical examination in G1.

Patency of porto-systemic shunts were evaluated by selective angiography of the superior mesenteric vein 24 hours after cavo-portal transposition in G2.

## Results

### Splenic transposition

The surgical procedure required a median of 10 min; the subcutaneous hematoma resulted by splenic decapsulation, gradually resolved in few days.

**Figure 1:**
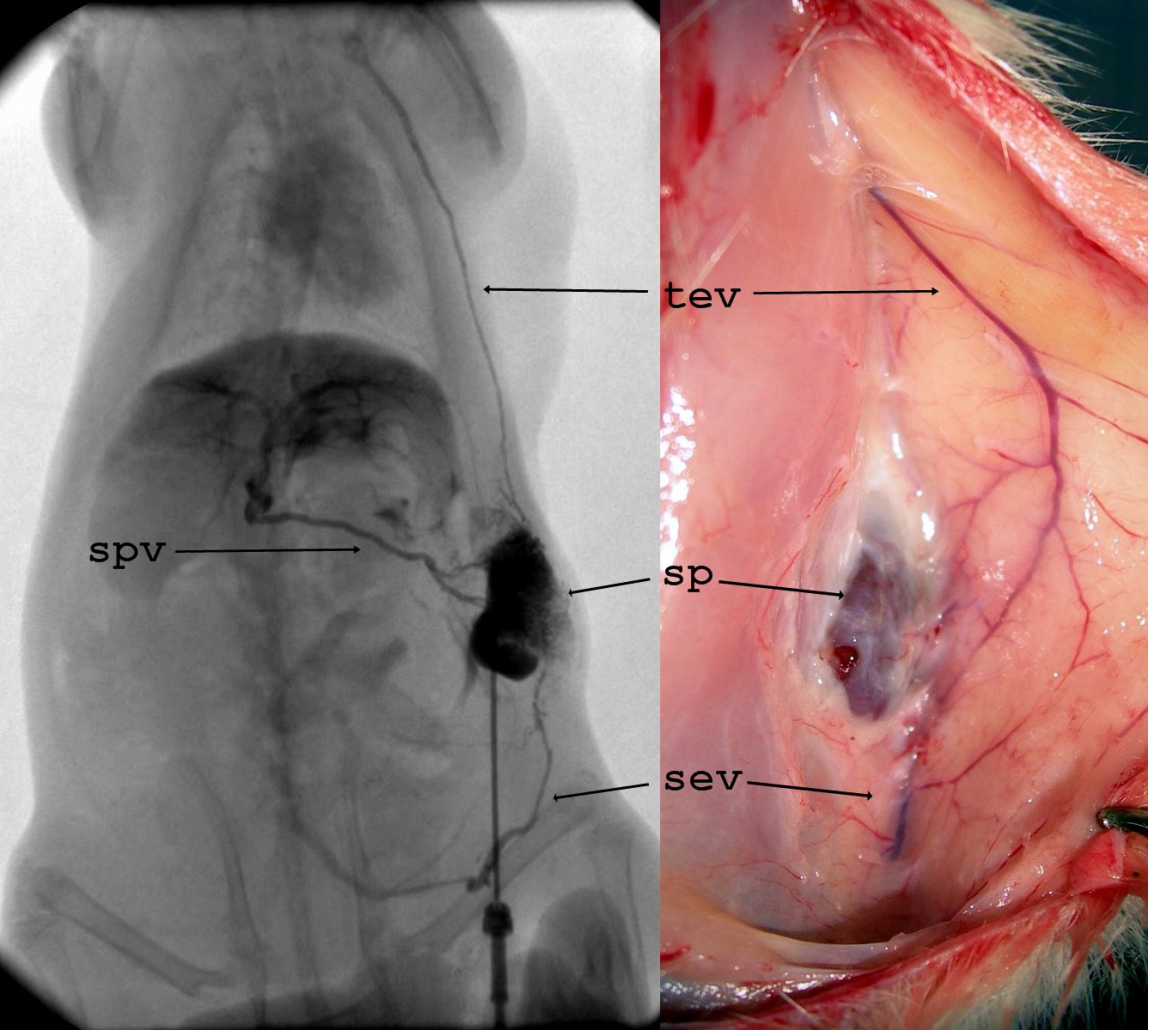
Left side: angiography by direct splenic puncture shows the physiological venous drainage through the splenic vein to the portal vein; neo formed capillaries connect the spleen with the superior vena cava through thoraco-epigastric vein, and the inferior vena cava through the superficial epigastric vein. Right side: macroscopical examination shows the collaterals connecting the transposed spleen with the thoraco-epigastric vein and the superficial epigastric vein. sp = spleen, spv = splenic vein, tev = thoraco-epigastric vein, sev = superficial epigastric vein.

After four weeks, neoformed vessels could be clearly seen by macroscopical examination from the splenic surface to the subcutaneous veins; angiography by direct splenic puncture showed the spleen drainage through the splenic vein and neoformed collaterals to the superior vena cava by thoraco-epigastric vein and to the inferior vena cava by superficial epigastric vein (Figure 1).

### Cavo-portal transposition

**Figure 2:**
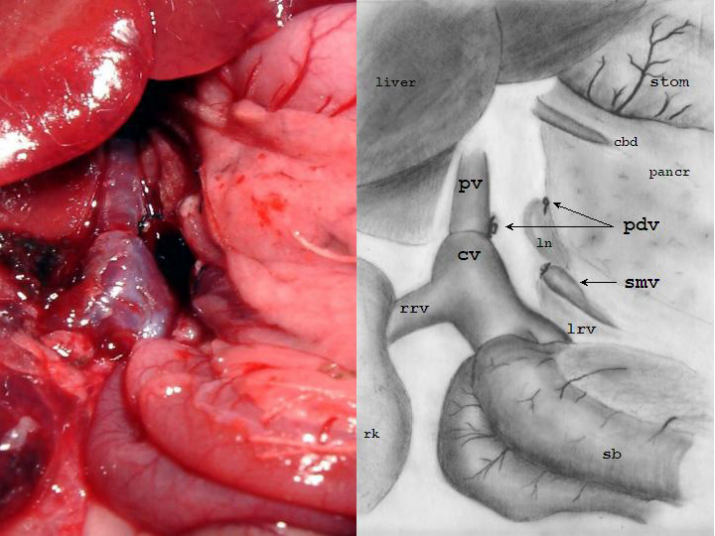
The liver is reperfused after cavo-portal transposition. pv = portal vein, cv = vena cava, smv = superior mesenteric vein, pdv = pancreatic-duodenal vein, rrv = right renal vein, lrv = left renal vein, cbd = common bile duct, ln = linfonode, rk = right kidney, sb = small bowel

The surgical procedure required a median of 50–60 minutes, with 10–15 min for cavo-portal anastomosis. Discrepancy of caliber between veins was overcome by small longitudinal incisions on the anterior and posterior wall of the portal vein. The final result is showed in figure 2.

**Figure 3:**
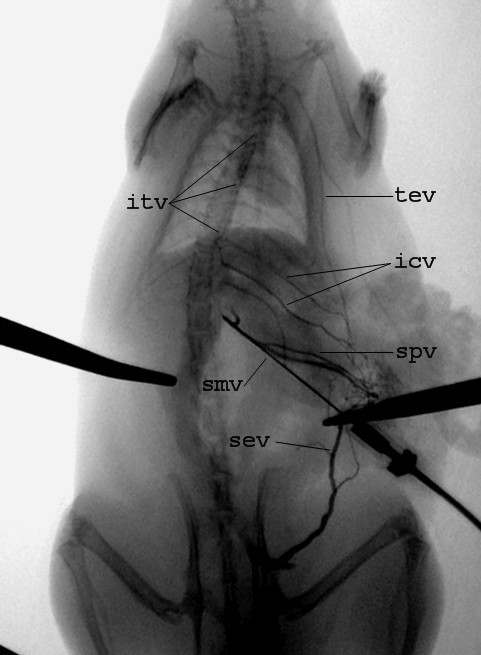
Selective mesenteric vein angiography after cavo-portal transposition: splanchnic flow is shunted to the vena cava system through the neo-formed porto-caval shunts. smv = superior mesenteric vein, spv = splenic vein, icv = intercostal veins, itv = internal thoracic vein, tev = thoraco-epigastric vein, sev = superficial epigastric vein.

All rats in G2 group survived 24 hours and underwent selective mesenteric angiography that showed total mesenteric blood flow diversion through the spleen to the superior and inferior vena cava (Figure 3). Macroscopical examination revealed normal bowel and liver appearance, absence of intestinal wall edema and no ascites.

## Discussion

Diffuse portal thrombosis is no longer a contraindication for liver transplantation.

Since the report of Tzakis et al. [4], the use of caval blood as portal inflow as been described as valuable strategy to performed liver transplantation in patient with diffuse splanchnic venous thrombosis [5-8,12,13]. In this setting, stabilized inframesocolic porto-systemic shunts are essential to guarantee splanchnic venous drainage in to the systemic circulation and to assure hepatotropic factors to the liver by systemic blood flow [7].

However, the clinical data available are heterogeneous, and further experimental studies are necessary to clarify the role of systemic blood inflow by porto-caval transposition on liver function [8,14].

In pre-transplant era, Child et al. reported normal liver function and normal liver regeneration after porto-caval transposition in dog, showing that physiological splanchnic blood flow could be replaced by systemic blood flow via the portal vein [11]. More recently the influence of systemic circulation on liver function has been investigated using a rat model of porto-caval transposition [9]. These studies suggested that the major determinant of the metabolic function of the liver is the amount of portal blood flow rather than its source.

However, these experimental models are challenging and they do not reproduce comparable clinical hemodynamic conditions, since the absence of splanchnic inframesocolic collaterals to the systemic circulation.

This new technique of cavoportal transposition, with previously induced porto-systemic shunts, is simple: it avoids the need of complex microsurgical reconstruction using vascular grafts from other animal, as previously described [9], and it leads to a quick and easy performance.

This model simulates the clinical hemodynamic condition since the porto-systemic shunts induced by subcutaneous splenic transposition represent the physiological collaterals induced by chronic portal thrombosis in man. It is effective: the induced shunts ensure an adequate splanchnic venous drainage as showed by angiography, and confirmed by the normal appearance of bowel and the absence of any signs of edema or ascites.

In addition, it can be used to study the liver regeneration and the ischemic precondition during cavo-portal transposition, indeed it is also suitable for whole or reduced liver transplantation with caval inflow.

## Conclusion

The technique we describe represents in our opinion the more realistic model of cavo-portal transposition in small animal: it is simple and effective, and it could be a valuable tool in studying the metabolic function of the liver with caval inflow in presence of total splanchnic flow diversion.

## Competing interests

The author(s) declare that they have no competing interests.

## Authors' contributions

SDD designed the study, performed the operations and prepared the manuscript. GB performed the radiological investigations. MG, FR participated in performing the operations and in preparing the manuscript. EA, DC, UV participated in the design of the study and coordination. All authors read and approved the final manuscript.

## Pre-publication history

The pre-publication history for this paper can be accessed here:


